# Fyn Mediates High Glucose-Induced Actin Cytoskeleton Reorganization of Podocytes via Promoting ROCK Activation* In Vitro*

**DOI:** 10.1155/2016/5671803

**Published:** 2016-01-06

**Authors:** Zhimei Lv, Mengsi Hu, Xiaoxu Ren, Minghua Fan, Junhui Zhen, Liqun Chen, Jiangong Lin, Nannan Ding, Qun Wang, Rong Wang

**Affiliations:** ^1^Department of Nephrology, Provincial Hospital Affiliated to Shandong University, Jinan 250021, China; ^2^Department of Obstetrics and Gynecology, Second Hospital of Shandong University, Jinan 250033, China; ^3^Department of Pathology, Medical School of Shandong University, Jinan 250012, China; ^4^Department of Nephrology, First Affiliated Hospital of Chongqing Medical University, Chongqing 400042, China

## Abstract

Fyn, a member of the Src family of tyrosine kinases, is a key regulator in cytoskeletal remodeling in a variety of cell types. Recent studies have demonstrated that Fyn is responsible for nephrin tyrosine phosphorylation, which will result in polymerization of actin filaments and podocyte damage. Thus detailed involvement of Fyn in podocytes is to be elucidated. In this study, we investigated the potential role of Fyn/ROCK signaling and its interactions with paxillin. Our results presented that high glucose led to filamentous actin (F-actin) rearrangement in podocytes, accompanied by paxillin phosphorylation and increased cell motility, during which Fyn and ROCK were markedly activated. Gene knockdown of Fyn by siRNA showed a reversal effect on high glucose-induced podocyte damage and ROCK activation; however, inhibition of ROCK had no significant effects on Fyn phosphorylation. These observations demonstrate that* in vitro* Fyn mediates high glucose-induced actin cytoskeleton remodeling of podocytes via promoting ROCK activation and paxillin phosphorylation.

## 1. Introduction

Advanced albuminuria, one of the signatures of diabetic nephropathy, causes progressive loss of renal function and leads to terminal renal impairment. Damage to podocytes, which is considered to be a possible early pathological marker for diabetic nephropathy, is important due to its role in causing albuminuria and glomerular damage [1–3]. In diabetic nephropathy, it is necessary to study the regulation of the actin cytoskeleton directly in podocytes because the dysregulation of the highly specialized podocyte actin cytoskeleton is closely associated with albuminuria [4, 5]. Fyn is a member of the Src family of kinases, and it responds to many stimuli to facilitate downstream signaling that regulates cell growth, adhesion, and motility [6, 7]. Earlier reports have demonstrated that Fyn associates with multiple intracellular substrates, such as focal adhesion kinase (FAK), paxillin, and *β*-adducin, to regulate cytoskeletal architecture and cell-cell interactions [8, 9]. It resides in podocytes and binds directly to and phosphorylates nephrin during podocyte differentiation and in response to injury, and this phosphorylation event results in polymerization of actin filaments [10–12]. Rho-associated coiled-coil forming protein kinase (ROCK) is a downstream effector of the small G protein Rho, which has been implicated in a variety of biological functions including cell contraction, migration, adhesion, and gene expression. The ROCK kinases phosphorylate a variety of substrates, which collectively lead to increased myosin ATPase activity and inhibition of the depolymerisation of actin and the assembly of stress fibers and focal adhesions; these events contribute to the reorganization of the actin cytoskeleton [11, 13]. Furthermore, it has been shown that ROCK may be a pivotal downstream target of Fyn for F-actin formation in fibroblasts exposed to lysophosphatidic acid [14]. The activation of ROCK in the kidney has been confirmed in models of diabetes both* in vitro* and* in vivo* [15–17], whereas inhibition of ROCK ameliorated the structural changes in the diabetic kidney together with a modest antiproteinuric effect [18]. In this study, we aimed to reveal the role of the Fyn/ROCK signaling pathway in podocyte injury that was induced by high glucose and its interactions with paxillin. We found that exposure of podocytes to high glucose led to a rearrangement of the filamentous actin (F-actin), accompanied by paxillin phosphorylation and increased cell motility. Both knockdown of Fyn by siRNA and inhibition of ROCK by Y27632 suppressed high glucose-induced F-actin rearrangement and paxillin phosphorylation. These findings demonstrated that high glucose-induced podocyte F-actin rearrangement was associated with the activation of Fyn/ROCK signaling pathway and the axis-mediated phosphorylation of paxillin.

## 2. Materials and Methods

### 2.1. Cell-Culture and Pharmacological Treatments

Conditionally immortalized mouse podocytes (a kind gift from Professor Peter Mundel) were cultured on type I collagen-coated dishes in RPMI1640 (HyClone, Logan, Utah, USA) supplemented with 10% fetal bovine serum (HyClone, Logan, Utah, USA), 100 U/mL penicillin, and 100 mg/mL streptomycin (HyClone, Logan, Utah, USA) under permissive conditions 33°C plus 10 U/mL mouse recombinant *γ*-interferon (Pepro Technology, London, UK). Podocyte differentiation was induced by maintaining podocytes on type I collagen-coated dishes at 37°C without *γ*-interferon for at least 14 days. Differentiated podocytes between passages 15 and 20 were incubated in serum-free RPMI1640 medium for 24 hours before being subjected to different treatments.

To investigate the effect of high glucose stimulation on podocytes, cells were exposed to low glucose medium (RPMI1640 medium containing 5.5 mM glucose and 10% fetal bovine serum) or high glucose medium (RPMI1640 medium containing 30 mM glucose and 10% fetal bovine serum) for 48 hours. Podocytes incubated in RPMI1640 with 5.5 mM glucose plus 24.5 mM mannitol and 10% fetal bovine serum for 48 hours were considered as the control for osmolarity. Podocytes were pretreated with 10 *μ*M Y27632 (Merck Chemicals Ltd., Nottingham, UK), an inhibitor reported to be specific for ROCK, for 30 minutes, and then incubated in high glucose medium for 48 hours to investigate whether ROCK is involved in high glucose-induced podocyte injury.

### 2.2. Fyn Gene Knockdown

RNA interfering technology was used during the investigation of Fyn as described below. Podocytes were prepared the day prior to transfection. Following incubation with Fyn siRNA or Cont-siRNA for 24 hours, podocytes were then either incubated in standard-glucose medium or high glucose medium for an additional 48 hours. Cells that were not transfected which were exposed to standard-glucose medium were considered to be controls. SiRNA oligonucleotides with the following sequences were designed: siRNA, 5′-CCT­GTA­TGG­AAG­GTT­CAC­AAT-3′; negative Cont-siRNA, 5′-TTC­TCC­GAA­CGT­GTC­ACG­T-3′. Transfection was performed in 24-well plates using 40 nM siRNAs in Lipofectamine 2000 (Invitrogen, Carlsbad, CA, USA). Fyn gene knockdown was confirmed by western blotting and quantitative real-time PCR.

### 2.3. Fyn Kinase Activity Assay

Podocytes were lysed in radioimmunoprecipitation assay (RIPA) buffer at 4°C for 10 minutes. Equal amounts of podocyte lysate were incubated with anti-Fyn antibody (Santa Cruz Biotechnology, CA, USA) at room temperature for 2 hours. Immunocomplexes were isolated using Protein A/G PLUS-Agarose (Santa Cruz Biotechnology, CA, USA) and recovered by boiling for 3 minutes in 1x electrophoresis buffer. Proteins were eluted with SDS-PAGE sample buffer and immunoblotted with anti-Src [PY418] (Invitrogen, CA, USA).

### 2.4. Measurement of ROCK Activity

The activity of ROCK was assessed by determining the phosphorylation state of myosin phosphatase targeting subunit 1 (MYPT1), a downstream target of ROCK, using anti-MYPT1 [PY853] antibody (Santa Cruz Biotechnology, CA, USA) and an appropriate secondary antibody (Jackson ImmunoResearch Laboratories, West Grove, PA) for immunoblotting.

### 2.5. Protein Isolation and Western Blotting Analyses

Cells were washed with PBS and lysed with 2x SDS-PAGE sample buffer. Next, 50 *μ*g of total protein was separated by SDS-PAGE and transferred to nitrocellulose membranes; the membranes were then blocked with 1% polyvinylalcohol in PBS containing 0.2% Tween 20 for 10 minutes and incubated at 4°C overnight with primary antibodies diluted to various concentrations in blocking buffer (5% or 1% skim milk in PBS-Tween (0.2% Tween 20)) targeted against the following target proteins: Fyn (1 : 100), Src [PY418] (1 : 500), paxillin [PY31] (1 : 5000), desmin (1 : 500), vimentin (1 : 500), synaptopodin (1 : 200), MYPT1 [PY853] (1 : 200), or *β*-actin (1 : 200). The membranes were then washed three times with PBS-Tween for 10 minutes and incubated with specific peroxidase-conjugated secondary antibodies diluted in blocking buffer (5% skim milk in PBS-Tween) for 2 hours at room temperature. Specific bands were detected using the ECL system and the Bio-Rad electrophoresis image analyzer (Bio-Rad Laboratories, Hercules, CA).

### 2.6. RNA Extraction and Quantitative Real-Time PCR

Total RNA was extracted from cells using the TRIpure Reagent (Takara, Dalian, China) according to the manufacturer's instructions. RNA samples were quantified by measuring optical absorbance at 260 and 280 nm. A260/A280 ratios ranged from 1.8 to 2.0, which indicated a high purity of the extracted RNA. The concentration of total RNA was calculated based on A260. Aliquots of total RNA (1.0 *μ*g each) from each sample were reverse-transcribed into cDNA according to the instructions of the PrimeScript RT Reagent Kit (Takara, Dalian, China). Quantitative real-time PCR was used to detect the specificity and knockdown efficiency of the Fyn siRNA and to determine the steady-state mRNA levels of synaptopodin and desmin and vimentin. Briefly, after reverse transcription of total RNA, cDNA was used as a template for the PCR reactions using gene-specific primer pairs. Amplification was performed using SYBR Premix Ex Taq Kit (Takara, Dalian, China) in the LightCycler 480 Real-Time PCR system (Roche Applied Science, Penzberg, Germany). The primers were purchased from Sangon Biotech Co., Ltd. (Shanghai, China). The designed sequences were as follows: synaptopodin: sense, 5′-CGG­AGA­ATC­AAA­ACC­CTC­AG-3′, antisense, 5′-CAG­GAC­ACT­GCC­ATC­AGA­CT-3′; desmin: sense, 5′-GTG­AAG­ATG­GCC­TTG­GAT­GT-3′, antisense, 5′-GCT­GGT­TTC­TCG­GAA­GTT­GA-3′; vimentin: sense, 5′-GAT­CAG­CTC­ACC­AAC­GAC­AA-3′, antisense, 5′-GCT­TTC­GGC­TTC­CTC­TCT­CT-3′; Fyn: sense, 5′-AAG­GAT­AAA­GAA­GCA­GCG­AAA­C-3′, antisense, 5′-TGC­GTG­GAA­GTT­GTT­GTA­GTTC-3′; *β*-actin: sense, 5′-GTG­GGC­CGC­TCT­AGG­CAC­CAA-3′, antisense, 5′-CTC­TTT­GAT­GTC­ACG­CAC­GAT­TTC-3′.

### 2.7. Immunofluorescence

Cells growing on glass slides were pretreated with different conditions and then fixed in 4% paraformaldehyde for 20 minutes followed by permeabilization in 0.3% Triton X-100 for 10 minutes. After preincubation with 5% bovine serum albumin to block nonspecific binding, cells were individually incubated with primary antibodies against synaptopodin (ProteinTech Group, Chicago, IL, USA), desmin (ImmunoWay, Newark, USA), vimentin (ProteinTech, Chicago, IL, USA), or paxillin [PY31] (Abcam Inc., Cambridge, MA, USA) at 4°C overnight. After washing, the slides were stained with Dylight 649-conjugated secondary antibodies (ZSGB-Bio, Beijing, China) and double-stained with DAPI to visualize nuclei. Cells were observed and images captured at randomly selected fields using an inverted phase fluorescence microscope (Leica, Wetzlar, Germany).

F-actin staining was in accordance with the procedure recommended by the dye manufacturer, and images were taken at randomly selected fields using an LSM780 confocal microscope (Carl Zeiss, Jena, Germany).

### 2.8. Transwell Migration Assay

Transwell cell-culture inserts (pore size 8 *μ*m; Corning Costar Corp., Cambridge, MA, USA) were rinsed once with PBS and placed in RPMI1640 with 10% fetal bovine serum in the lower compartment. The heights of the medium in the upper and lower compartments were maintained at similar levels; thus, bulk flow was not due to a hydrostatic pressure gradient. Podocytes pretreated with different conditions were harvested with trypsin and resuspended in serum-free RPMI1640 medium; the upper chambers were seeded with 1 × 10^4^ mL^−1^ cells, which were then allowed to attach for 6 hours. After incubation for 12 hours at 37°C, nonmigratory cells were removed from the upper surface of the membrane using cotton-tipped applicators, and migrating cells were fixed with 4% paraformaldehyde and stained with Hematoxylin. The number of migrating cells in the center of a membrane (one field) was counted using phase contrast microscopy (Leica, Wetzlar, Germany). The data presented denote the mean ± SD of 6 independent experiments.

### 2.9. Wound-Healing Assay

Confluent monolayers of podocytes seeded onto type I collagen-coated six-well plates were pretreated with different conditions and scraped with a 20 *μ*L pipette and then allowed to migrate for 12 hours. The percentage of wound closure was captured using an inverted phase contrast microscope (Leica, Wetzlar, Germany) and calculated using NIH ImageJ. Migratory rates were calculated as (*A* − *B*)/*A* × 100%, where *A* and *B* reflect the width of the wound at 0 or 12 hours, respectively. The data denote the mean ± SD of 6 independent experiments.

### 2.10. Statistical Analyses

The experiments were repeated at least three times. SPSS13.0 software was used for data analysis. Values are presented as the mean ± SD. Statistical significance was assessed using LSD *t*-tests and ANOVAs, and values of *P* < 0.05 were considered statistically significant.

## 3. Results

### 3.1. High Glucose Causes F-Actin Cytoskeleton Rearrangements and Injury to Podocytes

**Figure 1:**
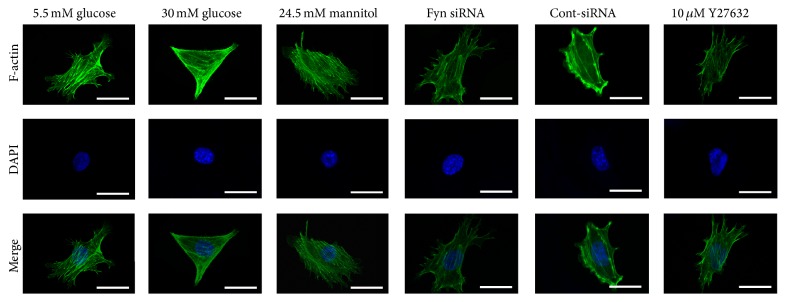
Reorganization of the F-actin cytoskeleton in podocytes by confocal microscopy. Podocytes under 5.5 mM glucose conditions (column 1) demonstrated that F-actin is distributed as obvious homogenous bundles that traverse the cell along the axis of the podocyte. 30 mM glucose stimulation for 48 hours (column 2) showed an assembly of F-actin in cortical regions, agminated F-actin along the cell periphery, and a slightly diffuse cytoplasmic distribution. Podocytes transfected with Fyn siRNA and then stimulated by 30 mM glucose for 48 hours (column 4) showed reversed F-actin distribution. Pretreatment with 10 *μ*M Y27632 for 30 minutes revealed similar F-actin amelioration (column 6). Podocytes that were exposed to high osmotic pressure (column 3) or transfected with Cont-siRNA (column 6) were considered as controls.

Podocyte foot processes (PFPs) are characterized by a podocome-like cortical network of branched actin filaments, which are linked to the glomerular basement membrane at focal contacts to modulate the permeability of the filtration barrier via changes in PFP morphology [19, 20]. In this study, morphological changes of podocytes under different conditions were captured using a confocal microscope. Podocytes under low glucose conditions demonstrated that F-actin is distributed as obvious homogenous bundles that traverse the cell along the axis of the podocyte (Figure 1). Exposure to high osmotic pressure for 48 hours caused no significant effect compared with incubation in a low glucose concentration. Podocytes stimulated with high glucose for 48 hours showed an assembly of F-actin in cortical regions, agminated F-actin along the cell periphery, and a slightly diffuse cytoplasmic distribution (Figure 1).

**Figure 2:**
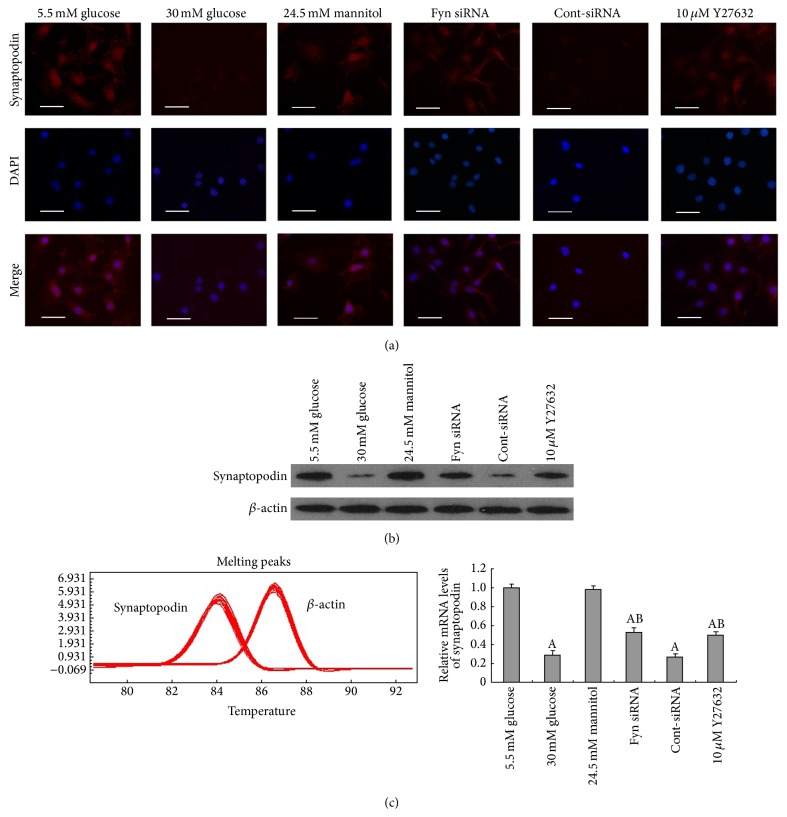
Alterations of protein and mRNA levels of synaptopodin in podocytes. Immunofluorescence (a), western blot (b), and real-time PCR (c) revealed that synaptopodin expression was markedly decreased following 48-hour incubation with 30 mM glucose (column 2) when compared with cells incubated in 5.5 mM glucose (column 1) on both protein and mRNA levels. Transfection with Fyn siRNA (column 4) or pretreatment with 10 *μ*M Y27632 for 30 minutes (column 6) partially reversed these alterations. Control podocytes were treated with 5.5 mM glucose plus 24.5 mM mannitol (column 3) or transfected with Cont-siRNA (column 5). Magnification: 400x. Values denote the mean ± SD; ^A^ *P* < 0.05 versus 5.5 mM glucose and ^B^ *P* < 0.05 versus 30 mM glucose.

**Figure 3:**
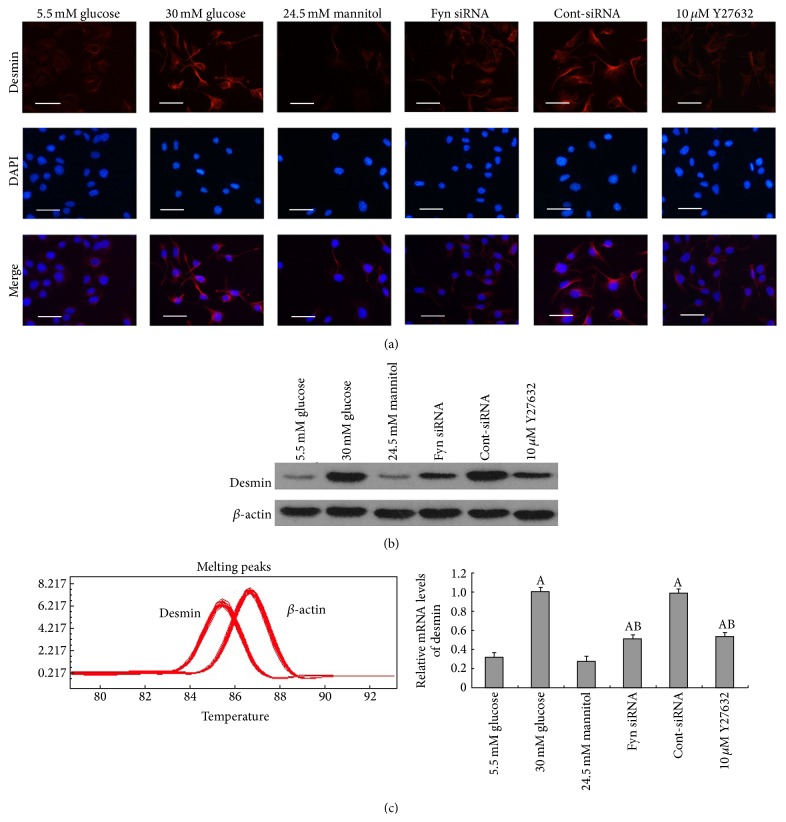
Changes of desmin on protein and mRNA levels in podocytes under different stimulations. Immunofluorescence (a), western blot (b), and real-time PCR (c) presented that desmin protein was increased markedly following incubation with 30 mM glucose for 48 hours (column 2), and the increase was partially inhibited by transfection with Fyn siRNA for 24 hours (column 4) or pretreatment with 10 *μ*M Y27632 for 30 minutes (column 6). Control podocytes were treated with Cont-siRNA (column 5) or 5.5 mM glucose plus 24.5 mM mannitol (column 3). Magnification: 400x. Values denote the mean ± SD; ^A^ *P* < 0.05 versus 5.5 mM glucose and ^B^ *P* < 0.05 versus 30 mM glucose.

**Figure 4:**
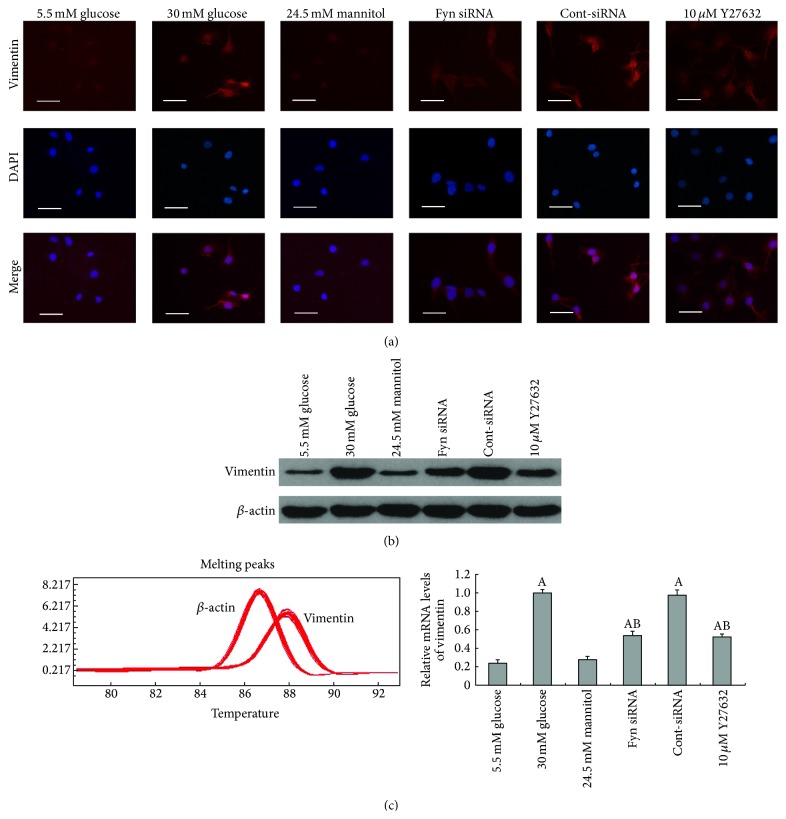
Alterations of vimentin on protein and mRNA levels in podocytes under different stimulations. Immunofluorescence (a), western blot (b), and real-time PCR (c) presented that vimentin expression was increased markedly following incubation with 30 mM glucose for 48 hours (column 2), and the increase was partially inhibited by transfection with Fyn siRNA for 24 hours (column 4) or pretreatment with 10 *μ*M Y27632 for 30 minutes (column 6). Control podocytes were treated with Cont-siRNA (column 5) or 5.5 mM glucose plus 24.5 mM mannitol (column 3). Magnification: 400x. Values denote the mean ± SD; ^A^ *P* < 0.05 versus 5.5 mM glucose and ^B^ *P* < 0.05 versus 30 mM glucose.

To fully characterize the changes in podocytes stimulated by high glucose, we further examined the expression of synaptopodin, desmin, and vimentin. Synaptopodin is an important actin-associated protein, a decrease of which has been confirmed as a remarkable phenomenon during podocytopathy [21–23]. Vimentin and desmin, which are known markers of podocyte injury [24, 25], were also assessed in this study. We observed that podocytes cultured for 48 hours in high glucose demonstrated a significant downregulation of synaptopodin compared with podocytes cultured in low glucose conditions, at both the protein and mRNA levels (Figure 2), whereas high osmotic pressure showed no obvious effects. The effects of high glucose on synaptopodin expression were further confirmed by immunofluorescence, where reduced fluorescence staining was observed (Figure 2). Moreover, significant increases in desmin and vimentin expression were also detected, at both the protein and mRNA levels following 48 hours of incubation in high glucose, compared with incubation with low glucose (Figures 3 and 4). Increased fluorescent staining further confirmed the high glucose-induced overexpression of desmin and vimentin in podocytes (Figures 3 and 4).

### 3.2. Fyn Is Involved in High Glucose-Induced Podocyte F-Actin Cytoskeleton Remodeling

**Figure 5:**
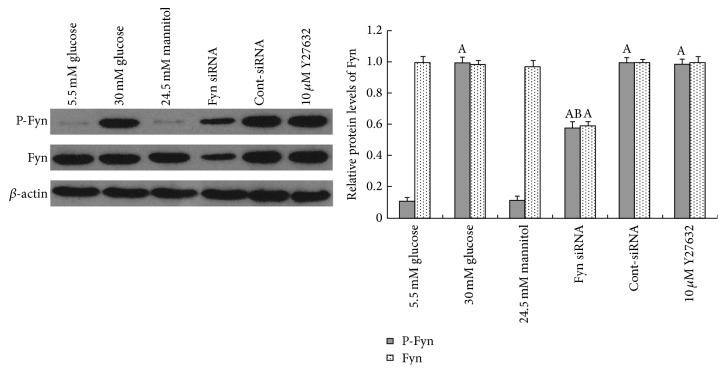
Western blot analysis of alterations to Fyn expression and Fyn activation in podocytes. Levels of activated Fyn exhibited a remarkable upregulation after 48-hour incubation with 30 mM glucose (lane 2) compared with cells incubated with 5.5 mM glucose (lane 1). Fyn siRNA significantly downregulated Fyn expression and activity (lane 4), whereas pretreatment with 10 *μ*M Y27632 for 30 minutes showed little effects on Fyn activation (lane 5). Control podocytes were treated with 5.5 mM glucose plus 24.5 mM mannitol for 48 hours (lane 3) or transfected with Cont-siRNA (lane 5). Values denote the mean ± SD; ^A^ *P* < 0.05 versus 5.5 mM glucose and ^B^ *P* < 0.05 versus 30 mM glucose.

**Figure 6:**
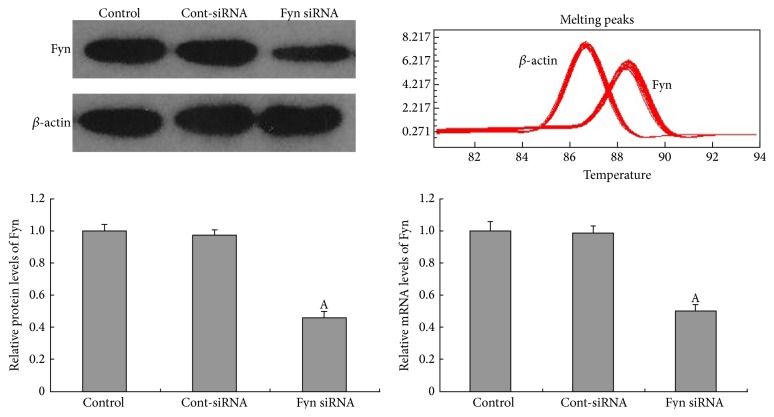
Fyn siRNA gene knockdown effects on Fyn expression in podocytes by western blotting and quantitative real-time PCR. A 24-hour transfection with Fyn siRNA notably reduced the expression of Fyn in cells treated with 5.5 mM glucose on both protein and mRNA levels (lane 3). Fyn expression was not significantly altered in podocytes transfected with Cont-siRNA for 24 hours (lane 2) compared with untransfected cells treated with 5.5 mM glucose (lane 1). Values denote the mean ± SD; ^A^ *P* < 0.05 versus 5.5 mM glucose.

As a member of the Src family of kinases, Fyn is known as a nonreceptor tyrosine kinase that responds to many stimuli to regulate the cytoskeleton and process formation. The activity of Fyn is regulated by intramolecular interactions and depends upon the phosphorylation state of two tyrosine residues, Y529 and Y418. Dephosphorylation of Y529 results in autophosphorylation at Y418 in the activation loop of the catalytic domain of Fyn, thereby stabilizing it in the active conformation [9, 26]. To detect activated Fyn, podocyte lysates were immunoprecipitated with anti-Fyn antibody followed by western blotting with anti-Src [PY418] antibody. As demonstrated by the western blotting (Figure 5), high glucose promoted Fyn activation in podocytes compared with cells incubated in low glucose conditions, with no alterations in total Fyn expression noted. To gain insight into the mechanism of the high glucose-induced podocyte F-actin cytoskeleton reorganization, we utilized a small interfering RNA (siRNA) to knock Fyn expression down. To validate the efficiency of the Fyn siRNA, podocytes were transfected with Fyn siRNA for 24 hours and then incubated in low glucose medium for another 48 hours. A significant reduction of Fyn at both the protein and mRNA levels was detected (Figure 6). Podocytes transfected with Fyn siRNA for 24 hours and then incubated under high glucose conditions for 48 hours were used to perform further experiments. The high glucose-induced F-actin cytoskeleton reorganization was markedly ameliorated by Fyn gene knockdown (Figure 1). Furthermore, after silencing Fyn expression with siRNA, the alterations to synaptopodin, desmin, and vimentin were also ameliorated (Figures 2, 3, and 4).

### 3.3. ROCK Is Involved in High Glucose-Induced Podocyte F-Actin Cytoskeleton Remodeling

**Figure 7:**
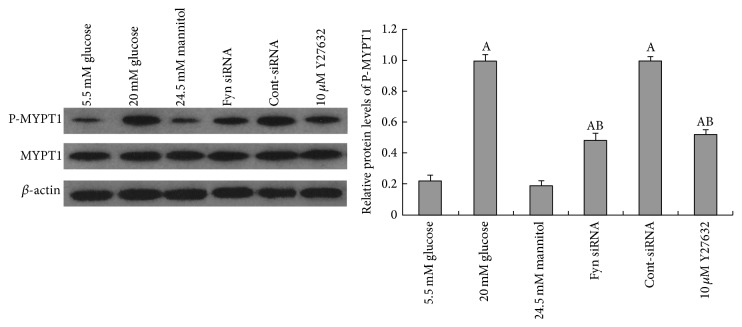
Western blotting analysis of alterations to ROCK activity in podocytes. ROCK activity was analyzed by determining the phosphorylation state of MYPT1, a downstream target of ROCK. The phosphorylation status of MYPT1 was significantly enhanced after 48-hour incubation with 30 mM glucose (lane 2) compared with cells incubated with 5.5 mM glucose (lane 1). Transfection with Fyn siRNA for 24 hours (lane 4) or pretreatment with 10 *μ*M Y27632 for 30 minutes (lane 6) decreased MYPT1 phosphorylation. Control podocytes were treated with Cont-siRNA (lane 5) or 5.5 mM glucose plus 24.5 mM mannitol (lane 3). Values denote the mean ± SD; ^A^ *P* < 0.05 versus 5.5 mM glucose and ^B^ *P* < 0.05 versus 30 mM glucose.

ROCK is a critical Rho effector in the regulation of F-actin, focal adhesion formation, cell morphology, and cell motility. The activation of Rho/ROCK in the kidney has been confirmed in models of diabetes both* in vitro* and* in vivo* [15–17]. Here, we demonstrated that podocytes cultured in high glucose for 48 hours showed increased ROCK activity compared with podocytes incubated in low glucose conditions, shown by the increase in phosphorylation of MYPT1 [27], a downstream target of ROCK (Figure 7). Knocking down Fyn with siRNA reversed the upregulation of ROCK activity stimulated by high glucose. Pretreatment with 10 *μ*M Y27632, an inhibitor specific to ROCK, for 30 minutes, inhibited effectively the high glucose-induced ROCK activation (Figure 7), with no significant alteration to total Fyn expression noted (Figure 5). The F-actin reorganization induced by high glucose was remarkably ameliorated by pretreatment with Y27632 (Figure 1). Moreover, the high glucose-induced alterations to synaptopodin, desmin, and vimentin were also partially reversed in the presence of Y27632, at both protein and mRNA levels (Figures 2, 3, and 4).

### 3.4. Under High Glucose Conditions, Paxillin Is Phosphorylated in Podocytes

**Figure 8:**
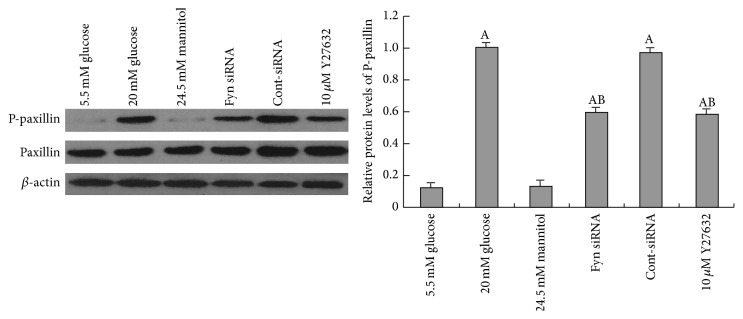
Western blotting analysis of the phosphorylation levels of paxillin in podocytes. The phosphorylation of paxillin (PY31) was significantly increased after a 48-hour incubation with 30 mM glucose (lane 2) compared with cells incubated with 5.5 mM glucose (lane 1), and no alterations in total paxillin expression were noted. Transfection with Fyn siRNA for 24 hours (lane 4) or pretreatment with 10 *μ*M Y27632 for 30 minutes (lane 6) partially decreased high glucose-induced paxillin phosphorylation. Control podocytes were treated with Cont-siRNA (lane 5) or 5.5 mM glucose plus 24.5 mM mannitol (lane 3). Values denote the mean ± SD; ^A^ *P* < 0.05 versus 5.5 mM glucose and ^B^ *P* < 0.05 versus 30 mM glucose.

**Figure 9:**
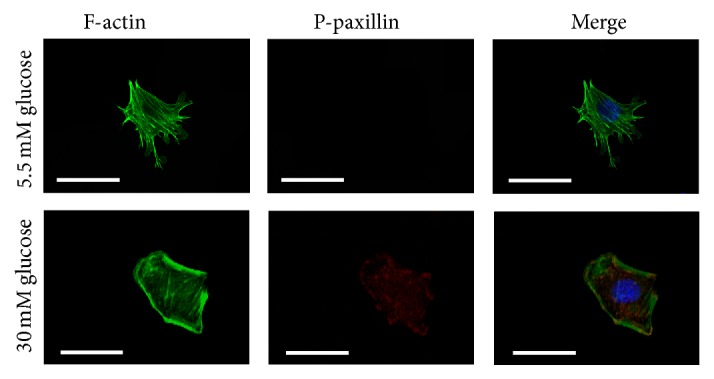
F-actin and paxillin expression by immunofluorescence. Under low glucose conditions (column 1), paxillin phosphorylation was undetectable and F-actin was distributed along the cell axis. After 48 hours of 30 mM glucose stimulation (column 2), the activation of paxillin was markedly upregulated with F-actin expressed at the cortical regions of the podocytes. Magnification: 400x.

Paxillin is a phosphorylation-dependent protein that links the intracellular actin cytoskeleton to integrin-rich adhesion sites. The phosphorylation of paxillin tyrosine residue 31 (Y31) is particularly important and prevalent in regulating downstream signaling and causes profound effects on the cytoskeleton, cell adhesion, and movement [28–30]. In this study, an increase in tyrosine phosphorylation of paxillin at Y31, which has been shown to be an effector of high glucose, was detected. Incubation with high glucose medium for 48 hours enhanced tyrosine phosphorylation of paxillin at Y31 compared with podocytes incubated in low glucose conditions or exposed to high osmotic pressure, without alterations of total paxillin expression; high glucose promoted paxillin phosphorylation at Y31 was markedly decreased by both Fyn knockdown and ROCK inhibition (Figure 8). Moreover, the immunofluorescence data showed that the distribution of phosphorylated paxillin was predominantly along the cell periphery and was associated with reorganized F-actin (Figure 9). These findings suggested that the Fyn/ROCK signaling pathway potentially interacted with paxillin in podocytes under high glucose conditions.

### 3.5. High Glucose Promotes Podocyte Spreading and Migration

**Figure 10:**
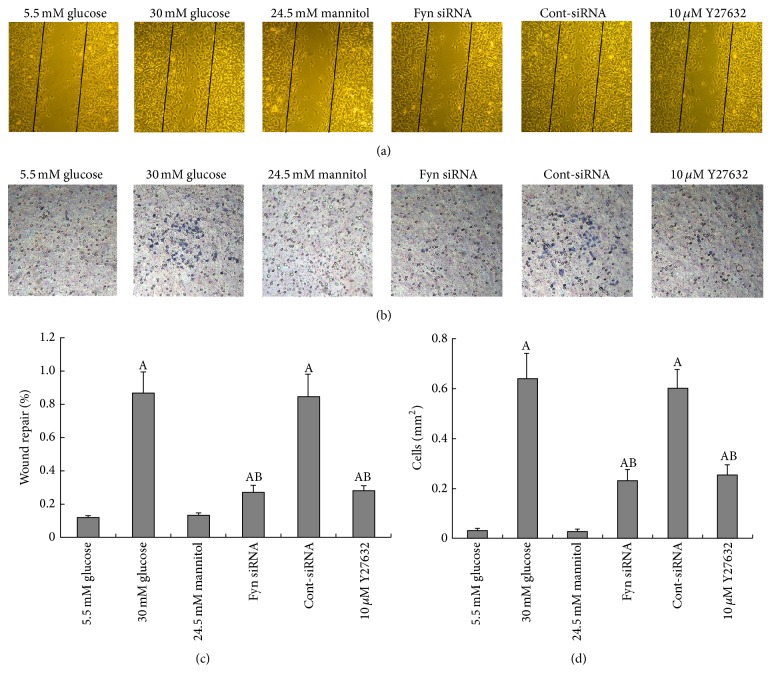
Podocyte motility by wound-healing assay and transwell migration assay. The wound-healing assay (a) and the modified transwell migration assay (b) were performed. Podocytes showed little migration when incubated in 5.5 mM glucose (column 1). Stimulation with 30 mM glucose-induced increased migration of podocytes (column 2), which was partially decreased by transfection with Fyn siRNA for 48 hours (column 4) or pretreatment with 10 *μ*M Y27632 for 30 minutes (column 6). Control podocytes were treated with Cont-siRNA (column 5) or 5.5 mM glucose plus 24.5 mM mannitol (column 3). Magnification: 200x.

To examine coordinated sheet migration, we next performed a scrap-wound-healing assay on a monolayer of podocytes. Compared with podocytes in standard-glucose conditions, high glucose increased the speed at which the podocyte monolayer filled a uniform-width scratch wound in. Both Fyn gene knockdown and inhibition of ROCK slowed high glucose podocyte wound healing (Figures 10(a) and 10(c)). Next, we used the modified transwell migration assay and wound-healing assay to test the motility of cultured podocytes in different conditions. Podocytes subjected to high glucose showed significantly increased cell migration through the transwell filters compared with those incubated in low glucose or high osmotic pressure conditions. This effect was reversed partially by both Fyn gene knockdown and inhibition of ROCK (Figures 10(b) and 10(d)).

## 4. Discussion

Podocytes possess a contractile structure, composed of abundant microfilaments that modulate glomerular filtration, and their structure is based on an actin cytoskeleton [4, 5]. Interference with cytoskeleton interactions with the slit diaphragm or defects in cytoskeletal components will cause foot process effacement, alterations in cell motility, and disruption of kidney filtration. Accumulating evidence has indicated that podocyte damage is a prerequisite for diabetic nephropathy, which, characterized by advanced albuminuria, has become one of the leading cause of end-stage renal disease [31]. It has been recently shown that effacement of podocyte foot processes occurs in diabetic nephropathy and that it is accompanied by a rearrangement of the actin cytoskeleton [3, 5, 32]. The podocyte cytoskeletal system includes actin regulatory proteins, such as *α*-actinin-4, synaptopodin, and the most important component called F-actin [4].

The actin cytoskeleton plays a crucial role in regulating the movement of cells via the highly dynamic and reversible polymerization of actin filaments, which provides the internal mechanical support that drives cell motility [4, 5]. In this study, under normal glucose conditions, podocytes showed obvious homogenous F-actin bundle distribution along the axis. Exposure to high glucose conditions for 48 hours led to the assembly of F-actin into cortical regions. And modified transwell migration assays and wound-healing assays revealed an increase in spreading and migration of podocytes cultured in high glucose conditions. Podocytes stimulated with high glucose showed significantly increased cell migration through transwell filters compared with cells subjected to normal glucose and high osmotic pressure conditions. Much work has demonstrated that increased podocyte motility may be an important aspect of podocytes depletion [4, 33, 34]. Therefore, it appeared that high glucose-induced dynamic rearrangement of the podocyte actin cytoskeleton, which subsequently promoted podocyte migration, which may initiate the depletion of podocytes and a vicious cycle of progressive glomerular damage.

Synaptopodin is an important marker of podocyte foot processes necessary for the maintenance of appropriate process structure and function in podocytes. A decrease in synaptopodin has been confirmed as an important phenomenon in podocytopathy [21, 22]. The results of this study demonstrate a significant decrease in synaptopodin expression at both mRNA and protein levels in podocytes cultured in high glucose conditions. Increased expression of desmin and vimentin, both observed in the present study, is associated with damage to podocytes in diabetic nephropathy [23, 24] and several other renal disease models [25, 35]. These observations revealed that high glucose-induced podocyte injury, characterized by a significant downregulation of synaptopodin together with an upregulation of desmin and vimentin at both mRNA and protein levels.

Fyn tyrosine kinase, a member of the Src family protein tyrosine kinases, has been suggested to be involved in cytoskeletal regulation and cell-process branching in a variety of cell types [6–9, 12]. Fyn is necessary for neuronal synaptic regulation, and overexpression of Fyn exacerbates neurodegeneration [36]. Since podocytes and neurons share many structural and molecular features, a similar mechanism may be involved in the modulation of podocyte processes [37]. Previous studies have shown that, in podocytes, Fyn directly binds to nephrin and phosphorylates the latter at tyrosine residues during podocyte differentiation [12]. In our study, we found that the high glucose stimulation led to the activation of Fyn, accompanied by a rearrangement of F-actin bundles. Gene knockdown of Fyn by its specific siRNA ameliorated this high glucose-induced Fyn activation and F-actin remodeling, suggesting that Fyn is a triggering factor for F-actin cytoskeletal rearrangement under high glucose conditions.

ROCK, the best studied downstream target of RhoA, is involved in a myriad of signaling cascades including the regulation of stress fibers, focal adhesion formation, and cell motility. It is also a pivotal controller of the actin cytoskeleton during cell morphogenesis and migration [11, 13, 14, 16]. In the kidney, ROCK activation was found in models of diabetes both* in vitro* and* in vivo* [15–17]. And inhibition of ROCK helped ameliorate structural changes in the kidney together with a modest antiproteinuric effect [18]. Our results presented that ROCK could be activated by high glucose stimulation, which occurred concomitantly with the rearrangement of F-actin. Pretreatment of Y27632, the ROCK inhibitor, showed decreased activity of ROCK and partially reversed F-actin remodeling. Prior studies have suggested that, in fibroblasts, Fyn kinase may act as an upstream factor of ROCK in the pathway governing the cytoskeletal changes and process remodeling [14, 38]. In our study, we found that gene knockdown of Fyn remarkably inhibited ROCK activation, with reversed F-actin remodeling, whereas pretreatment of Y27632 showed no significant effects on total Fyn or phosphorylated Fyn expression, suggesting that, under high glucose conditions, Fyn promoted F-actin cytoskeletal remodeling via activation of ROCK.

Paxillin serves as a phosphorylation-dependent signaling scaffold, functioning in integrating and disseminating signals that affect cell adhesion, motility, and actin cytoskeletal remodeling [27]. A recent study suggested that lipopolysaccharide-induced podocyte injury was accompanied by increased paxillin phosphorylation and cell spreading [39]. In this study, we observed significantly upregulated paxillin phosphorylation in podocytes after high glucose treatment, which occurred concomitantly with F-actin bundles redistribution. Extensive research on cancer has documented that Fyn associates with intracellular substrates like FAK, paxillin, and *β*-adducin to regulate cytoskeletal architecture and cell-cell interactions [8, 40, 41]. In human umbilical vein endothelial cells, the activation of the ROCK signaling pathway was followed by tyrosine phosphorylation of paxillin, which further led to actin reorganization [42]. Inhibition of ROCK, however, could attenuate bombesin-induced increase in tyrosine phosphorylation of paxillin in Swiss 3T3 cells [43]. Our findings provided credible data that the upregulation of paxillin activity by high glucose was markedly abolished by Fyn gene knockdown or pretreatment of ROCK inhibitor, demonstrating that high glucose-stimulated phosphorylation of paxillin was in close association with Fyn/ROCK signaling pathway in cultured podocytes. Kleveta et al. found that LPS-induced phosphorylation of paxillin was responsible for the actin reassembly in macrophage and reassembly-associated cell motility. It is hypothesized that reorganization of F-actin may be regulated by phosphorylated paxillin under high glucose conditions, which was triggered by the activation of Fyn/ROCK signaling pathway.

The dynamic regulation of the podocyte cytoskeleton is paramount for the appropriate function of kidney filtration barrier. In the current study, we sought further confirmation that the reorganization of the F-actin cytoskeleton, along with paxillin phosphorylation and increased cell motility, is a consequence of Fyn/ROCK signaling pathway activation in high glucose-induced podocyte injury, represented by alterations to synaptopodin, desmin, and vimentin expression. These observations shed light on one possible mechanism responsible for podocyte dysfunction in diabetes mellitus and may facilitate the development of novel strategies for treating diabetic nephropathy by targeting cytoskeletal rearrangement in podocytes.
